# Supplementary material for: The genome sequence of a weevil, Polydrusus cervinus (Linnaeus, 1758)
Source: Wellcome Open Res. 2023 Dec 5;8:563. [Version 1] doi: 10.12688/wellcomeopenres.20414.1 (PMC11153995; doi:10.12688/wellcomeopenres.20414.1)
Supplement: Peer review history

# Reviewer report: Reviewer response for version 1

**DOI:** 10.21956/wellcomeopenres.22602.r83488

**Published:** 2024-06-05

**Authors:** Guilherme B. Dias

**Affiliations:** 1 Department of Cell and Molecular Biology, Uppsala University (Ringgold ID: 8097), Uppsala, Uppsala Country, Sweden

The authors report the genome sequence and gene annotation of the weevil *Polydrosus cervinus*. The resulting assembly is of very high quality based on contiguity, with scaffolds representing near-complete chromosomes (its unclear which chromosomes are T2T). The assembly is also complete both in terms of conserved endopterygota single-copy genes (99.2%), as well as overall k-mers (100% compared to the HiFi sequencing reads). The authors use well established and documented methods for assembly and curation, and also for gene annotation.

 The one point where the provided information is perhaps not complete is regarding the gene annotation procedure. This was performed as part of Ensembl's Rapid Annotation pipeline and not described in detail here. The link provided ( https://rapid.ensembl.org/Polydrusus_cervinus_GCA_935413205.1/Info/Index) does have some additional information, but lacks detail on which database versions and exact taxonomic divisions were used as protein evidence to run BRAKER2. I believe this information could be easily added to the methods.

Are sufficient details of methods and materials provided to allow replication by others?

Partly

Is the rationale for creating the dataset(s) clearly described?

Yes

Are the datasets clearly presented in a useable and accessible format?

Yes

Are the protocols appropriate and is the work technically sound?

Yes

Reviewer Expertise:

genome assembly, genome annotation, comparative genomics, repetitive dna

I confirm that I have read this submission and believe that I have an appropriate level of expertise to confirm that it is of an acceptable scientific standard.

# Reviewer report: Reviewer response for version 1

**DOI:** 10.21956/wellcomeopenres.22602.r75706

**Published:** 2024-05-28

**Authors:** Manee M Manee

**Affiliations:** 1 King Abdulaziz City for Science and Technology, Riyadh, Saudi Arabia

Barclay et al. conducted this study to present the weevil *Polydrusus cervinus* genome sequence as part of the Darwin Tree of Life Project, aiming to sequence all named eukaryotic species in the Atlantic Archipelago. This work generated a high-quality genome assembly of 713.4 megabases, including 11 chromosomal pseudomolecules and the mitochondrial genome, with 23,058 protein-coding genes identified. The assembly achieved a BUSCO completeness of 99.2%, utilizing Pacific Biosciences single-molecule HiFi long reads and meticulous manual curation. This effort contributes significantly to biodiversity research and the understanding of eukaryotic genomes, highlighting the importance of this study in the broader scientific community. The study could benefit from comparing its genome with those of similar species or other weevils to gain insights into evolutionary relationships.

Are sufficient details of methods and materials provided to allow replication by others?

Yes

Is the rationale for creating the dataset(s) clearly described?

Yes

Are the datasets clearly presented in a useable and accessible format?

Yes

Are the protocols appropriate and is the work technically sound?

Yes

Reviewer Expertise:

bioinformatics, genomics, and evolutionary biology

I confirm that I have read this submission and believe that I have an appropriate level of expertise to confirm that it is of an acceptable scientific standard.
